# Adverse drug reaction reporting practice and associated factors among medical doctors in government hospitals in Addis Ababa, Ethiopia

**DOI:** 10.1371/journal.pone.0227712

**Published:** 2020-01-21

**Authors:** Solomon Shiferaw Nadew, Kidanemariam G/Michael Beyene, Solomon Worku Beza

**Affiliations:** 1 USP/PQM, Addis Ababa, Ethiopia; 2 GAMBYMedical and Business College, Addis Ababa, Ethiopia; University of Oxford, UNITED KINGDOM

## Abstract

**Introduction:**

Adverse drug reactions (ADRs) are global public health problems. In its severe form it may cause hospital admission, morbidity and mortality. Early reporting of suspected ADRs to regulatory authorities is known to be appropriate measure toinsure health and safety of public form such adverse drug reaction of drugs. In Addis Ababa, there is limited information on ADR reporting practices among medical doctors. Hence, this study aimed to assess ADR reporting practices and associated factors among doctors in government hospitals in Addis Ababa.

**Methods:**

An institution based cross-sectional mixed-methods study design was used. Data werecollected from 407 doctors using self-administered questionnaire and five key informants using semi-structured questionnaire from October 01 to December 31, 2017. Binary logistic regression and thematic analysis methods for quantitative and qualitative data analysis were used respectively.

**Results:**

Only 94(27.4%) of doctors had ever reported ADRs to national pharmacovigilance center. The study showed that sex (AOR = 3.51, 95% CI: 1.76–7.03), level ofeducation (AOR = 5.01, 95% CI: 2.23–11.28), work experience (AOR = 4.59, 95% CI: 1.21–17.40), existence of ADR reporting form (AOR = 3.96, 95% CI: 1.07–14.61) and reporting to respective marketing authorization holders (AOR = 21.41, 95% CI: 5.89–77.88) were significantly associated with ADR reporting practices. Poor awareness and training on risk of under-reporting, feeling that reporting is minor, absence of appropriate reporting tools, delay and/or absence of feedback on reported ADRs, overly burdened doctors, negligence, fear of legal liabilityand communication gap were cited by key informants as barriers for reporting practice.

**Conclusions:**

Adverse drug reaction reporting practice among doctors wasfound to be low. Sex, level of education, work experience, existence of reporting form and reporting to marketing authorization holderswere significantly associated with ADR reporting practice. In addition, there are gaps in availabilities of guidelines, reporting systems and structure, pre-service and in-service training, and awareness of doctors on impact of reporting. Hence, improving access to ADR reporting form, decentralize safety monitoring system, and conducting awareness training on ADR reporting are essential to improve the ADR reporting practice.

## Introduction

Quality, safety and efficacy assured medicines are essential for patients’ health. Medicines have undergone through pre-clinical and clinical studies to prove its safety and efficacy before marketing authorization is granted. However, they have been tested on restricted numbers and types of patients for a limited time under strict protocols. In most cases, the vulnerable such as pregnant women, children and elderly have often been excluded from these clinical studies. This may create unfeasible conditions to detect rare adverse drug reactions. Problems related with medicines safety can emerge from real-life medication use related to inadequate labeling, packaging and product information. Post-marketing monitoring is therefore an important step to detect medicine-related problems that were not possible to identify during the pre-marketing phases [1].

Adverse drug reactions, as a response to drugs, is noxious and unintended, and which occurs at doses normally used in man for prophylaxis, diagnosis, therapy of disease, or for the modification of physiological functions [2].

Public health and safety from adverse drug reactions is of paramount importance in clinical practices. Adverse drug reactions are major global public health problems. It varies in magnitude; in its severe form may cause morbidity, mortality, hospital admissions, risk of readmission, increases length of hospital stay and other negative impacts [3–5]. Besides, adverse drug reaction impacts patients’ quality of life and the hospital system [6].

Globally, pharmacovigilance (PV) is one of the main tools used to improve patient safety and care through detecting problems associated with medicines use, and assessing their benefits, effectiveness, harms and risks to prevent injuries and maximize therapeutic outcomes[7].Adverse drug reactions underreporting is the main challenge of pharmacovigilance [8]. Health care providers are main source of suspected ADR reports to appropriate medicine regulatory agencies[7, 9].Studies showed that ADR under-reporting is directly linked toknowledge, attitude and practice of healthcare professionals and availability of ADR reporting systems[8, 10, 11]. In addition, false perception about ADRs, personal and professional conflicts, absence of efficient and well-established pharmacovigilance system resultedin under-reporting of ADRs encountered by the health professionals[10, 11].

A study conducted in tertiary care centre in United Arab Emirates revealed that 54.8% of clinicians were not aware of existence of pharmacovigilance centre and very few clinicians (14.3%) had reported ADRs to pharmacovigilance centre. The common factor that discourages ADR reporting in this study was lack of knowledge on how to report ADRs (71%). With respect to who to report ADRs, the study indicated that 97.6% opined clinicians, 81% stated nurses and pharmacists, and 42.9% believed patients (42.9%) could report ADRs [12].

A study conducted among medical doctors in India showed that spontaneous reporting rate was 19.1%. Inadequate risk perception about newly marketed drugs (77.9%), lack of clarity of information on ADR reporting form (52.9%), lethargy (42.7%), insufficient training to identify ADRs (41.2%), lack of awareness about existence of pharmacovigilance programs (30.9%) and ADR monitoring center (19.1%) were major factors responsible for ADR under-reporting [10].

A study conducted among medical doctors in Pakistan indicated that majority of respondents (88%) were aware of ADRs; 31.5% were aware of existence of pharmacovigilance programs; 7.5% had access to ADR reporting system; and only 9.7% were informed about the availability of ADR reporting system. The study also reported that physicians (64%) were considered the most qualified health professionals to report ADRs [13]. Another cross-sectional study done in Kuwait showed that 74.6% medical doctors had identified ADRs during their daily practice while only 34.2% had ever reported ADRs [14].

In Africa, the uniqueness of ADRs from the African population should be appreciated and put in context[15, 16]. A total of 41,870 ADRs were reported from 27 countries of sub-Saharan Africa in 1992 to 2013 [15]. In 2015,vigiBase data showed, 25 (of 35) of sub-Saharan African countries had less than 10 individual case safety reports per million person year [16]. It is recognized that Africa has some of its known limitations on drug safety monitoring such as under reporting, limited information and lack of denominators for ADR reports [16].

A cross sectional survey conducted among medical doctors in Accra, Ghana showed that 27.4% of medical doctors had received training on drug safety monitoring and ADR reporting systems which later improved the ADR reporting. In addition, the study revealed that 59.5% of medical doctors had encountered patients with suspected ADRs although only 20% of them had reported by completing spontaneous adverse drug reaction reporting form[17].

A study done in Gondar University Teaching Hospital and Felegehiwot Hospital, Amhara region, Ethiopia revealed that 52.9% of health professionals had encountered severe ADRs though not reported to national regulatory agency. This study also indicated that the major obstacles for adverse drug reaction reporting were lack of information (97.2%), absence of training on ADR reporting systems (83.3%), lack of knowledge on pharmacovigilance programs (77.8%) and absence of reporting tools such as reporting forms, phone or fax number (66.7%)[18]. A similar cross sectional study conducted among health care professionals in public hospitals in Amhara region, Ethiopia, indicated that none of the respondents know about national ADR monitoring guidelines, 65.8% of the respondents had insufficient knowledge about ADR reporting systems and 16.2% of the respondents had ever reported ADR encountered during their professional practice [19]. Another cross sectional study conducted in Gondar, Amhara region from 2013 to 2015 showed that 815 chemotherapy-related ADRs were identified from 203 patients’ medical files and were not reported [20].

A study conducted in Jimma University specialized hospital, Ethiopia, on HIV patients under highly active antiretroviral therapy (HAART) showed that 70.8% of them had developed ADRs [21].

Adverse drug reaction monitoring is one of the main priority agendas of the government of Ethiopia. In Ethiopia, all suspected ADRs including medication errors and product quality defects has to be reported[1]. Healthcare professionals are obliged to be vigilant to detect and report suspected ADRs to Ethiopian Food, Medicine and Healthcare Administration and Control Authority, EFMHACA (the national medicine regulatory authority where the national pharmacovigilance center of Ethiopia resides). This will help the Authority to take action in preventing or minimizing occurrence of medicine-related injuries [1, 22]. In addition, patients who suspect and experience adverse drug events are expected to report to health care professionals and the national medicine regulatory authority [1, 23].

However, in Ethiopia despite the potential risks of adverse drug reactions, to date there are about 600 ADR cases reported per year to the national pharmacovigilance center. The available reports at the national medicine regulatory authority are very low as compared to the World Health Organization’s recommended adverse drug reaction reporting rate, which is 200 reports per million population per year[20, 21, 24–27]. Adverse drug reaction reporting experiences among medical doctors in Addis Ababa are not well known and the reasons are not properly explored. There is gap between ADR cases generated from patient’s medical files and the reported data available at the national medicine regulatory authority[20, 21, 24].

The findings of this study will provide information to policymakers and relevant stakeholders to devise strategies and appropriate interventions to improve ADR reporting practice so as to prevent risks associated with adverse drug events, and improve therapeutic outcomes and quality of patient care.

Hence, this study aimed to assess adverse drug reaction reporting practices and associated factors among medical doctors in government hospitals in Addis Ababa, Ethiopia.

## Methods

### Study design and setting

An institution-based cross-sectional mixed-methods study design was conducted to assess the practice of ADR reporting and associated factors among doctors working in government hospitals with concurrent design to triangulate the quantitative data with the qualitative data. Data were collected from October 01 to December 31, 2017, in Addis Ababa.

Addis Ababa is the diplomatic capital of the African Union and capital city of Ethiopia. It has ten sub-cities and 116 administrative districts. The city has an estimated population of 3,384,569 [28]. During the study period, there were 13governmenthospitals, 26 private hospitals [29], 92 functional government health centers, 378 pharmacies, 278 drug shops and 777 different types of clinics in Addis Ababa [30]. According to the data collected from EFMHACA and Addis Ababa Food, Medicine and Healthcare Administration and Control Authority and hospitals, there were 1,846 doctors in Addis Ababa of which1,519and 327 of them were working in governmental and private hospitals respectively.

### Study population

The study participants were doctors working in the selected government hospitals in Addis Ababa with a minimum of six months work experiences. The key informants were selected based on the experience and responsibility in their respective organizations in relation to monitoring of adverse drug reactions.

### Sample size and sampling procedure

The sample size for the quantitative study was calculated using a single population proportion formula assuming 50% of the doctors are practicing to report ADRs with 95% confidence interval (CI), 5% margin of error and 10% non-response rate. The final sample size was 422.

All government hospitals were included in the study. Simple random sampling technique was used to select study participants. Using proportional allocation, 422 doctors were selected from the hospitals.

For the qualitative study, sample size was determined in advance to select the key informants from relevant organizations/stakeholders:EFMHACA, Ministry of Health, Addis Ababa Health Bureau, USP/PQM, TikurAnbesa Specialized Hospital. Purposive sampling technique was used to select key informants. One key informant from each organization was selected. Total of five key informantswere selected for interviewbased on their experience and responsibility in the respective organizations in relation to monitoring of adverse drug reactions.

### Data collection tools, procedures and quality assurance

The quantitative data were collected using self-administered structured questionnaire which was adapted from other studies and contextualized into local situations[20, 21].Before the actual data collection, the self-administered questionnaire was pre-tested on 5% randomly selected medical doctors from government hospitals and necessary amendments were made. The participants in the pre-test were not included during the actual data collection.

The questionnaire consisted of questions related to socio-demographic factors, knowledge, attitude, availability of reporting system (such as ADR reporting forms), institutional factors and ADR reporting practice. To assess knowledge about ADR reporting practices seven multiple choice questions related with ADR reporting practice were used. Each question was coded, computed and scores were dichotomized into knowledgeable (participants who scored ≥70% on knowledge-based questions) and not knowledgeable (participants who scored <70% on knowledge based questions). Doctors’ attitude regarding ADR reporting was assessed using 10 attitude-based questions and scored with five Likert scales (0–4). All attitude-based questions were coded, computed and the scores were categorized into favorable attitude (participants who scored ≥70% on attitude-based questions) and unfavorable attitude (participants who scored <70% on attitude-based questions). The collected data were checked for consistency and completeness before analysis. Finally, Epi-Info version 7.2.1.0 was used to control and manage errors resulting from data entry process.

For the qualitative study, semi-structured open-ended interview questionnaire with probing questions was prepared and used to collect the qualitative data from key informants. The principal investigator (the corresponding author of this study) carried out one-on-one, face-to-face, in-depth interviews. The interview was done inlocal language (Amharic) and any ambiguities raised from the interviewee were cleared at the time of the interview. The interviews were audio recorded and notes were taken properly. The participants were interviewed at the time and location of their choice. The average duration of the interviews was about 20minutes.

### Data management and analysis

The collected quantitative data were entered into Epi-Info version 7.2.1.0 and analyzed using SPSS version 23.0 software. Participants’ socio-demographic characteristics, knowledge, attitude and ADR reporting practice were described using relevant descriptive statistics. Bivariate analysis was done at 25% level of significance to screen out potentially significant independent variables[31]. The association between the dependent and independent variables were analyzed using Binary Logistic regression model. The adequacy of the final multiple Binary Logistic regression model was checked using the Hosmer and Lemeshow goodness-of-fit test and the final model fitted to the data well (p-value = 0.375). Results were expressed as a crude, adjusted odds ratio, and 95% confidence interval. Variables with p-value < 0.05were considered as statistically significant.

For the qualitative study, the audio records and notes of interviews were transcribed using non-verbatim transcription technique. Two experienced reviewers read the transcript and gave comments for the analysis before data synthesis and report writing. The transcribed scripts were intensively read to identify key themes and the data were synthesized thematically. The data were analyzed manually, and thematic analysis method was used. To check the accuracy of the translation one of the recording was translated and transcribed by a bi-lingual expert and compared with the primary work. Furthermore, the findings of the study were communicated to the key informants for authenticity of the transcripts and interpretation.

### Ethics approval and consent to participate

The study was approved by GAMBY Medical and Business College (GAMBY, IRERC, 2017), and Addis Ababa Health Bureau Ethics Review Board (Ref. No: AAHB/2732/227). An official support letter was granted by the Ethiopian Food, Medicine and Healthcare Administration and Control Authority to conduct the study. Prior to data collection, permission was also obtained from all hospitals selected for the study. In addition, written consent was secured from all study participants and the key informants.

## Results

### Socio-demographic characteristics

Among 422 medical doctors, 407 participated in the studymakinga response rate of 96%. Majority, 290 (71.3%) of the study participants were male. The mean age of the doctors is 30.6±5.84 with range of 23 years to 58 years and 162 (39.8%) of them were in the age of 28–32 years old. With respect to the level of education, 336 (82.6%) were general practitioners while 71 (17.4%) were specialists in different specialization. Two hundred fifty-eight (63.4%) of the study participants had one to three years of work experience **(Table 1).**

**Table 1:** Socio-demographic characteristics of doctors in Addis Ababa, 2017 (n = 407).

Variables	n (%)
**Sex**	
Male	290(71.3)
Female	117(28.7)
**Level of education**	
General practitioners	336(82.6)
Specialists	71(17.4)
**Age (in years)**	
23–27	144(35.4)
28–32	162(39.8)
>32	101(24.5)
**Work experience (in years)**	
1–3	258(63.4)
4–6	101(24.8)
>6	48(11.8)

### Knowledge on ADR reporting practice

The study showed that 123 (30.2%) of the study participants have never heard about the existence of ADR reporting system in Ethiopia. Almost half of the respondents (49.6%) did not know theexistence of National Guidelines for ADR Monitoring. In addition, two hundred ninety (71.3%) of doctors did not know how to report ADR cases to responsible body. From those who knew how to report ADRs, only 51 (43.6%) of them indicated that the responsible organization for monitoring ADR in Ethiopia is EFMHACA and 70 (59.8%)of the medical doctors did not know the existence of ADR reporting form. Three hundred sixty-one (88.7%) of doctors were not knowledgeable about ADR reporting practices **(Table 2).**

**Table 2:** Knowledge of ADR reporting practice among doctors in Addis Ababa, 2017 (n = 407).

Variables	n (%)
Ever heard about existence of ADR reporting system in Ethiopia	
Yes	284(69.8)
No	123(30.2)
Knowledge about existence of National ADR Monitoring Guidelines	
Yes	205(50.4)
No	202 (49.6)
Knowledge about how to report ADRs	
Yes	117 (28.7)
No	290 (71.3)
Organization responsible for monitoring ADR reports	
Ministry of Health	37 (31.6)
EFMHACA	51(43.6)
Universities	2 (1.7)
Ethiopian Public Health Institute (EPHI)	10(8.5)
Ethiopian Pharmaceutical Association	8 (6.8)
I don’t know	9(7.7)
Knowledge about existence of ADR reporting form (n = 117)	
Yes	47(40.2)
No	70(59.8)
Professionals responsible in ADR reporting (n = 117)	
Doctors	14(12)
Nurses	0
Pharmacy professionals	3(2.6)
All health professionals	97(82.9)
I don’t know	3(2.6)
Knowledge about ADR reporting practice	
Knowledgeable	46 (11.3)
Not knowledgeable	361(88.7)

**Fig 1:**
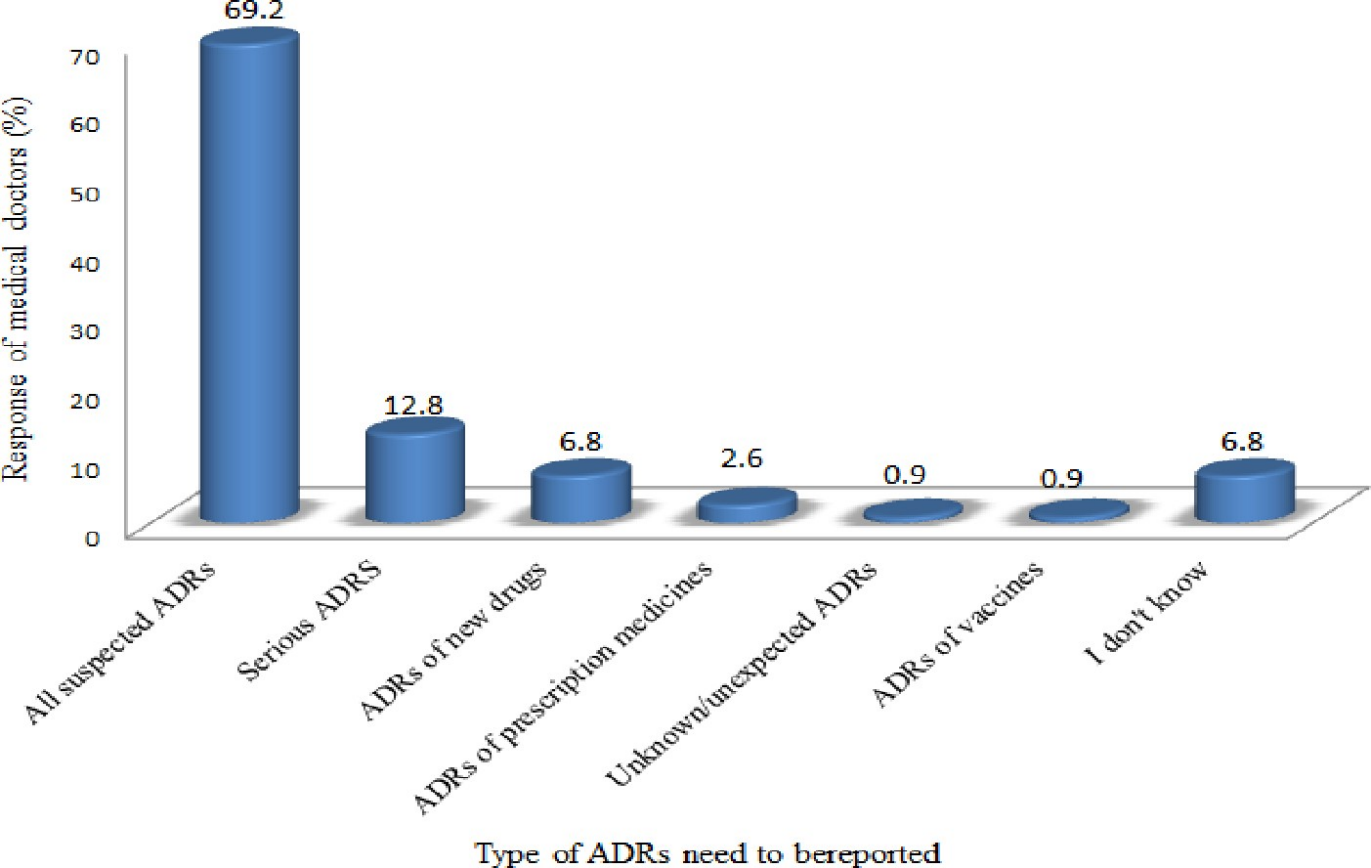
Doctors’ response on type of ADRs to be reported in Addis Ababa, 2017 (n = 117).

Regarding to the type of ADRs that need to be reported, majority (81/117, 69.2%) of the respondents showed that all suspected ADRs should be reported while 15 (12.8%) of them indicated that only serious ADRs should be reported **(Fig 1).**

### Pre-service and in-service training on ADR reporting

The study showed that 319(78.4%) and 376(92.4%) of the doctors were not provided pre-service and in-service training on ADR reporting systemsand how to report ADR respectively. Only 31(7.6%) of doctors got in-service training, of which12(2.9%), 7(1.7%), 5(1.2%), 6(1.5%) and 1(0.2%)were trained by EFMHACA, non-governmental organization, respective hospitals, EPHI and Health Bureau respectively.

### Attitude towards ADR reporting practices

Most of doctors (388/407, 95.3%) agreed that ADR reporting is the duty of all health professionals. However, 381 (93.6%) of doctors believed that ADRs need to be sure before reporting despite the need to report all suspected ADR cases encountered by the health professionals. In addition, 144 (35.4%) of doctors did not agree on the need to report all suspected ADR cases. More than half of doctors (51.4%) believed that fear of legal liability affected ADR reporting practices. The study revealed that286 (70.3%) of doctors hadunfavorable attitude while 121(29.7%) of them had favorable attitude towards ADR reporting practices **(Table 3).**

**Table 3:** Attitude towards ADR reporting practices among doctors in Addis Ababa, 2017 (n = 407).

Variables	n(%)				
	SA*	Agree	Undecided	Disagree	SD*
ADR reporting is duty of health professionals	142 (34.9)	246 (60.4)	12 (2.9)	6 (1.5)	1(0.2)
ADRs need to be sure before reporting	109(26.8)	272(66.8)	22(5.4)	4(1.0)	0(0)
ADR report improves patient’s safety	130(31.9)	261(64.1)	16(3.9)	0(0)	0(0)
All suspected ADRs should be reported	68(16.7)	195(47.9)	52(12.8)	83(20.4)	9(2.2)
ADR reporting trends identify relatively safe drugs	76(18.7)	252(61.9)	53(13.0)	20(4.9)	6(1.5)
ADR reporting creates workload	4(1.0)	51(12.5)	63(15.5)	211(51.8)	78(19.2)
ADR reporting is not important for healthcare system	2(0.5)	14(3.4)	29(7.1)	229(56.3)	133(32.7)
Reporting of ADR affects patient’s confidentiality issues	5(1.2)	30(7.4)	136(33.4)	179(44.0)	57(14.0)
A single ADR report brings no difference	3(0.7)	42(10.3)	77(18.9)	170(41.8)	115(28.3)
Fear of legal liability affects ADR reporting	17(4.2)	192(47.2)	128(31.4)	61(15.0)	9(2.2)

* SA represent as “strongly agree” and SD as “strongly disagree”

### ADR reporting form

The study indicated that only few (31/407, 7.6%) of respondents claimed that ADR reporting form existed in their hospital. However, 60(14.7%) of the respondents did not know the existence of the form in their hospital and 316(77.6%) of the respondents claimed that such form did not exist in their hospital.

From those respondents who claimed ADR reporting form exist in their hospital, 14(45.2%) of the respondents got ADR reporting form from Drug and Therapeutic Committee (DTC) and/or Drug Information Center(DIC) in the hospital and 9(29.0%) of them got from hospital administration. Six (19.3%) of the respondents claimed that EFMHACA has provided the ADR reporting form to doctors and 2(6.5%) got from ADR focal person in the hospital.

### Institutional factors

Only 39(9.6%) of the respondents indicated that the hospital they were working in had a systemincluding availability of responsible department and standard procedures to report ADRs to the national pharmacovigilance center. Seventy (47.2%) of doctors revealed that ADR reporting is one of the roles of DTC/DIC and 51(12.5%) of doctors responded that their hospitals have linkage to national pharmacovigilance center. However, very few doctors (27/407, 6.6%), indicated that their hospitals received support from national pharmacovigilance center. In addition, 246(60.4%) of the study participants claimed that the Marketing Authorization Holder (MAH) were not willing to receive ADR reports on their specific products **(Table 4).**

**Table 4:** Institutional factors that affect ADR reporting practice of doctors in Addis Ababa, 2017 (n = 407).

Variables	n (%)
Existence of systems in hospital to report ADR	
Yes	39(9.6)
No	298(73.2)
I don’t know	70(17.2)
ADR reporting considered as role of DTC/DIC	
Yes	70(47.20)
No	54(36.2)
I don’t know	25(16.8)
Existence of ADR focal person in hospitals	
Yes	19(4.7)
No	183(45.0)
I don’t know	68(16.7)
Linkage between hospital and national pharmacovigilance center	
Yes	51(12.5)
No	266(65.4)
I don’t know	90(22.1)
Support from pharmacovigilance center to hospitals*	
Yes	27(6.6)
No	306(75.2)
I don’t know	74(18.2)
Reporting ADRs to respective MAH	
Yes No	38(9.3) 309(75.9)
I don’t know	60(14.7)
Willingness of MAH to receive ADR reports of their own medicines	
Yes	61(15.0)
No	246(60.4)
I don’t know	100(24.6)

* Support included provision of training and ADR reporting form etc.

### ADR reporting practice

The study showed that 343(84.3%) of medical doctors encountered ADR cases during their professional carrier of which 299(87.2%) of the doctors recorded the ADR cases encounteredcases in the patients’ medical records and only 94(27.4%) of them had ever reported ADR cases to responsible bodies during their professional carrier (**Table 5**).

**Table 5:** ADR reporting practice among doctors in Addis Ababa, 2017 (n = 407).

Variables	n(%)
Encounter ADRs during professional carrier	
Yes	343(84.3)
No	64(15.7)
Record ADR on patient medical records	
Yes	299(87.2)
No	44(12.8)
Ever report ADRs during your professional carrier	
Yes	94(27.4)
No	249(72.6)
Number of ADR cases reported during professional career	
Only once	23 (24.5)
2–3 times	27(28.7)
More than three times	44(46.8)
Ever presented ADR cases at morning meeting	
Yes	149(43.4)
No	194(56.6)

**Fig 2:**
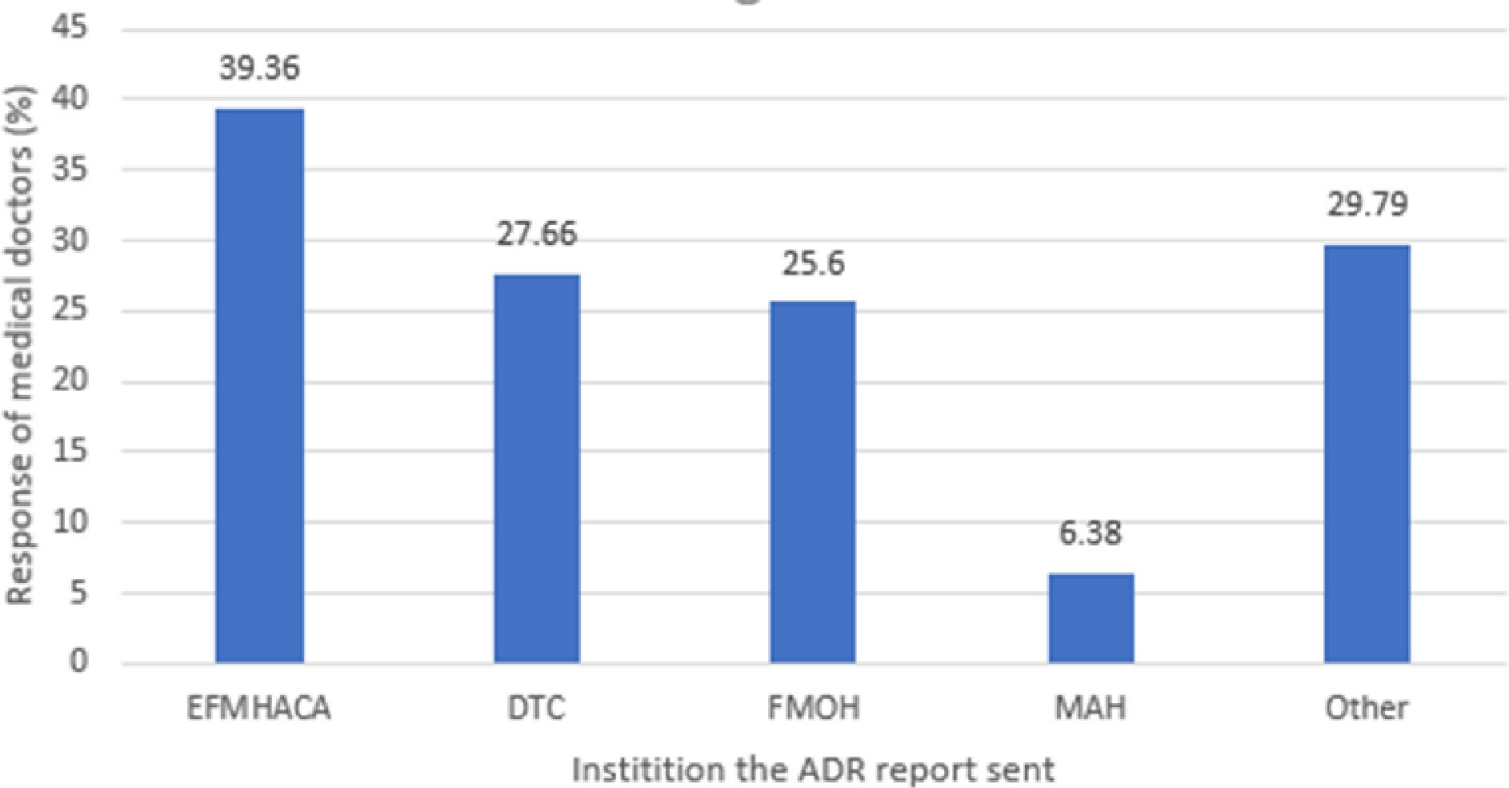
Doctors’ reponse on where to ADRs encountered in Addis Ababa, 2017 (n = 94). Others include Addis Ababa Health Bureau, EPHI and NGOs.

The study showed that only 39.36% of the doctors who had ever encountered ADR cases reported ADRs to EFMHACA (**Fig 2**).

### Factors associated with ADR reporting practice

In the bivariable analysis; age, sex, level of education, work experience, knowledge on ADR reporting, pre-service and in-service trainingon ADR reporting, existence of ADR reporting form and procedure on ADR reporting, reporting to hospital DTC/DIC, linkage and support of hospital to/by PV center, and report of ADR cases to MAH were found to be statistically significant with ADR reporting practice. In multivariable analysis only sex, level of education, work experience, existence of ADR reporting formand reporting to MAH were significantly associated with ADR reporting practice.

In this study, sex was significantly associated with ADR reporting practice. Female doctors were 3.5 times more likely to report ADR cases to national PV center as compared to male doctors (AOR = 3.51, 95% CI: 1.76–7.03).

Level of education was significantly associated with ADR reporting practice. Specialists were five times more likely to report ADR cases to national PV center as compared to general practitioners (AOR = 5.01, 95% CI: 2.23–11.28).

Work experience was significantly associated with ADR reporting practice. Doctors having more than six years of work experience were 4.6 times more likely to report ADR cases to national PVcenter as compared to doctors having one to three years of work experience (AOR = 4.59, 95% CI: 1.21–17.40).

Availability of ADR reporting form in hospitals was significantly associated with ADR reporting practice. Doctors who claimed the availability of ADR reporting form in their hospitals were four times more likely to report ADR cases to national PV center as compared to those doctors who did not claim the existence of ADR reporting form(AOR = 3.96, 95% CI: 1.07–14.61).

Reporting ADR cases to MAH was significantly associated with ADR reporting practices. Doctors who reported ADR cases to respective MAH were 21 times more likely to report ADR cases to national PV center as compared to those doctors who did not report ADR cases to MAH (AOR = 21.41, 95% CI: 5.89–77.88) **(Table 6).**

**Table 6:** Bivariable and multivariable analysis of factors affecting ADR reporting practice among doctors in Addis Ababa, 2017(n = 343).

Variables	ADR reporting Practice		COR (95% CI)	AOR (95% CI)	P-Value
	Yes	No			
Age (in years)					
23–27	26	97	0.83(0.46–1.49)	0.70(0.33–1.48)	0.385
28–32	33	102	1	1	
>32	35	50	2.16(1.21–3.88)	0.53(0.18–1.55)	0.344
Sex					
Male	52	189	1	1	
Female	42	60	2.54(1.54–4.19)	**3.51(1.76–7.03)**	<0.001*
Level of education					
General practitioner	55	222	1	1	
Specialist	39	27	5.83(3.29–10.34)	**5.01(2.23–11.28)**	<0.001*
Work experience (in years)					
1–3	44	167	1	1	
4–6	23	66	1.32(0.74–2.36)	1.28(0.58–2.83)	0.534
>6	27	16	6.41 (3.17–12.92)	**4.59(1.21–17.40)**	0.025*
Knowledge on ADR reporting					
Not Knowledgeable	72	229	1	1	
Knowledgeable	22	20	3.50(1.81–6.78)	1.14(0.40–3.24)	0.813
Pre-service training on ADR reporting					
No	58	202	1	1	
Yes	36	47	2.67(1.58–4.50)	1.82(0.88–3.24)	0.106
In-service training on ADR reporting					
No	75	242	1	1	
Yes	19	9	8.76(3.55–21.64)	2.03(0.50–8.35)	0.325
Existence of ADR reporting form in hospital					
Yes	17	8	6.34(2.62–15.37)	**3.96(1.07–14.61)**	0.039*
No	66	197	1	1	
I didn’t know	11	44	0.75(0.36–1.53)	0.46(0.13–1.59)	0.218
Existence of system in hospitals to report ADR					
Yes	21	12	5.7(2.65–12.29)	0.89(0.27–2.96)	0.842
No	58	189	1	1	
I didn’t know	15	48	1.02(0.53–1.95)	1.05(0.32–3.43)	0.939
Linkage between hospitals and PV center					
Yes	27	17	5.58 (2.81–11.06)	2.67(0.75–9.50)	0.130
No	49	172	1	1	
I didn’t know	18	60	1.05 (0.57–1.95)	0.460(0.10–2.21)	0.332
Existence of hospital support from PV center					
Yes	13	9	4.52(1.84–11.08)	0.66(0.12–3.64)	0.634
No	62	194	1	1	
I didn’t know	19	46	1.29(0.71–2.37)	4.21(0.92–19.26)	0.064
Medical doctors report ADR to respective MAH				1	
Yes	28	5	22.29(8.21–60.53)	**21.41(5.89–77.88)**	<0.001*
No	52	207	1	1	
I didn’t know	14	37	1.51(0.758–2.991)	1.29(0.39–4.27)	0.672
Willingness of MAH to receive ADR reports					
Yes	25	27	3.23(1.71–6.11)	0.82(0.27–2.52)	0.726
No	45	157	1	1	
I didn’t know	24	65	1.29(0.73–2.29)	1.86(0.70–4.93)	0.210

*Statistically significant at 5% level of significance multivariable analysis

### Qualitative findings

A total of five key informants were interviewed. The key informants were pharmacists by profession and assumed roles as directors, team leaders and experts. Their age ranges from 37–45 years with 10 to 20 years of work experiences in various organizations of which four are males and one female.

The key informants were asked about the overall situations, guidance, systems and knowledge of adverse drug reaction reporting practiceamong medical doctors working in government hospitals. Key themes emerged from the analysis were (i) ADR reporting practice, (ii) ADR reporting systems and structure, (iii) Knowledge of medical doctors towards ADR reporting, (iv) attitude of medical doctors on ADR reporting, (v) guidelines and formsof ADR reporting, and (vi) reasons for ADRunder reporting. Results of the qualitative data were presented in detail as follows.

#### ADR reporting practice

All study participants pointed out that the understandings of the impact of reporting a single suspected ADR case by medical doctors were low. The same holds true with other health professionals. One key informant augmented as:

“….Currently, the PV center received not more than 600 ADR reports per year. The numbers of ADR reports at national level were very low as compared to the expected World Health Organizationminimumstandards (200 reports per one million populations per year). Considering 90 million population of Ethiopia, I expected about 18, 000 ADR reports per year which is too far from the existing reporting practice” (ARP01).

#### Reasons for under reporting practice

The participants indicated that there were under-reporting of ADR cases and many barriers were identified for adverse drug reactions under-reporting. Medical doctors did not report due to a number of reasons including poor awareness and training on risk of under-reporting, feeling that reporting is minor, absence ofappropriatereporting tools, absence of focal person in the hospital, lack of encouragement, delay and/or absence of feedback on the reported ADRs from the national PV center, overly burdened medical doctors, fear of legal liability, and communication gap among patient, medical doctors and the PV center.

#### Knowledge on ADR reporting

The key informants thought that knowledge on ADR reporting among medical doctors was inadequate. This included awareness on reporting systems and toolsincluding why ADR reporting is needed, to whom to report, where to report, which adverse event is to be reported and what are the tools used to report and what information to gather on adverse events etc. One of the respondents supported this as:

“…..the knowledge on reporting ADR cases was low. Medical doctors managed ADRs when encountered in the hospital. I said this boldly because in other countries ADR is considered as one of the top ten causes of death but in our country, there is no data on the implication of ADRs. Hence we didn’t know the existing situation about ADRs” (ARP05)

#### Attitude towards ADR reporting

Two of the key informants indicated that there was negligence among medical doctors in reporting of ADR cases encountered. Some of the medical doctors had wrong perceptions on whom to report ADR cases. Since pharmacists manage pharmaceuticals, some medical doctors believed that ADR reporting is the responsibility of pharmacy professionals which severely affected the ADR reporting practice of medical doctors. One of the respondents expressed his view as follows:

“…. As pharmacy personnel have direct involvement on drugs, medical doctors perceived that ADR reporting is the responsibility of pharmacy professionals only. This might be due to lack of awareness among medical doctors on the consequences of ADRs and failed to recognize their role to manage drug related risks. This needs serious attention from relevant stakeholders” (ARP05).

Another key informant showed that medical doctors assumed that they need to be sure before reporting ADR cases. However, EFMHACA required health professionals to report any suspected ADR cases of any kind. This is expressed as:

“…when ADR happened, majority of medical doctors want to be sure and confirm whether this ADR is occurring due to medicine or by diseases or any other cause. However, confirmation of ADR is not requirement and being suspicious is sufficient to report ADR cases” (ARP01).

#### ADR reporting system

All key informants agreed that there wasformalsystem to report ADR cases to the national PV center. Despite its existence, the system is weak and unable to encouragemedical doctors to report ADR cases and capture sufficient reports as expected. The Authority has introduced mechanisms to report ADR cases such as ADR reporting form, email account and free toll-8482. One of the respondents showed that the national minimum health facility standards required health professionals including medical doctors to detect and report ADR cases as one of the daily activities. In addition, health facilities are required to assign focal persons to organize reports generated from the facilities and report to national PV center.

One key informant re-enforced the weakness of the available ADR reporting system. Medical doctors assumed to detect ADR cases mostly from inpatients. He mentioned as:

“…..Patients might encounter ADR cases at home. There was no suitable system for medical doctors to detect and report ADR cases from patients that encountered at home. Even though the patients wantto be consulted and report what was happening in their health due to drugs, most patients faced difficulties to access doctors.However, in-patients took medicines under the follow-up of nurses and/or medical doctors, which makes it easier to detect ADR cases. Despite this, I did not believe that detected ADR cases are reported to national PV center”(APR03).

Another key informant mentioned that the national PV center were not equipped with electronic reporting systems which could facilitate reporting of ADR cases. The authority needs to consider establishing electronic reporting system including mobile applications. Besides, the forum available at EFMHACA to manage reported ADR cases was not effective in investigating and providing feedbacks to reported cases (ARP01).

#### ADR guidelines and reporting form

Some of the key informants indicated that due to shortage of ADR reporting forms, it was not accessible to health facilities and medical doctors, which was not encouraging to report ADR cases to the Authority. EFMHACA tried to avail the reporting forms through its branch offices and regional health bureaus. It also sent acknowledgements letter together with additional reporting forms when received ADR reports. In addition, the respondents stated that EFMHACA had developed guidelines for drug safety monitoring but it was not accessible to end users including medical doctors. As claimed by the participants, the health facilities including medical doctors did not use the guidelines as guidance to report ADR cases.

#### Structure and organization

Effective structure and organization are crucial for ADR reporting. Some of the key informants indicated that the current structure was not healthy, decentralized and accessible to the health facilities and health professionals. The exiting PVcenterwas at federal level. One of the key informants emphasized that:

“………… Currently there is only one national PV center in Ethiopia. Decentralization of the center will facilitate easy access to reporting forms and guidelines. Training activities including fact-to-face discussions can be provided easily with low costs. Establishing regional centers at university hospitals could serve and facilitate to ease ADR reporting by medical doctors” (APR06).

## Discussion

The present study assessed adverse drug reaction reporting practice and associated factors among doctors working in government hospitals in Addis Ababa, Ethiopia. The study found that few doctors had ever reported ADRs to national PV center. Majority of doctors had unfavorable attitude towards ADR reporting practice and did not have the knowledge on ADR reporting practice.

The study showed that 84.3%of doctors encountered ADR cases during their professional carrier. This is consistent with studies conducted in Nigeria and Malaysia [32, 33]but higher as compared to study conducted in Ghana[17].The difference,in the finding of this study with study conducted in Ghana, might be due to difference in study setting, healthcare setting, time and awareness about the importanceofreporting a single adverse drug reaction.

The findings of this study showed that only 27.4% of doctors reported adverse drug reactions to national PV center. The qualitative results also indicated that there was under reporting practice of adverse drug reactions by doctors. The result was consistent with studies conducted in India, Kuwait and Nigeria [14, 32, 34] but higher compared to studies conducted in Ghana and India [10, 17, 35]and lower than reports indicated by the study conducted in United States of America [36]. The reasons for under reporting might be absence of getting feedbacks from the national PV center on reported ADRs, lack of awareness on impact of reporting a single ADR, need to be sure before reporting, uncertainty on how to report, negligence, fear of legal liability and inefficient reporting system and tools in the country. The qualitative results of this study substantiated these findings.

It is interesting to note that under reporting of adverse drug reaction is common in most countries. Different studies declared that lack of awareness about reporting of ADR cases, weak system for reporting, absence of teaching about ADR reporting in undergraduate curriculum, absence of periodic reinforcement of ADR monitoring in internship and postgraduate studiesandlack of proper tools for reporting werethe main driving factors for under reporting of adverse drug reactions[8, 37–39]. In addition, ignorance of the detected ADR, insecurity (fear of legal liability) and indifference whether to report the ADR detectedwere also among the factors associated with under-reporting of adverse drug reactions[38].

The study found that sex, level of education, work experience, existence of ADR reporting form and ADR reporting to MAH were significantly associated with ADR reporting practice. Female doctors were3.5 times more likely to report ADRsas compared to male doctors. This might be females have more aptitude to report adverse drug reactions they encounter as compare to the male counterpart[40]. Furthermore, the females might have positive perception towards pharmacovigilance and ADR reporting[41]. However, this requires further investigation.

Level of education was significantly associated with ADR reporting practice.Doctors with specialization (specialists) are five times more likely to report ADR casesas compared to general practitioners. This is similar with a study done in Ghana [17] and Egypt[42]. The possible reasons might be because specialists had got in service ADR trainings and better experiences. Furthermore specialists were more knowledgeable on PV and ADR reporting than the general practitioners[42], This provided them better position to report ADRsencounteredto the national PV center.

Doctors having more than six years of work experience were4.6 times more likely to report ADR cases as compared to doctors who had one to three yearsof work experience. This might be due to the fact that longer work experience increased exposure to different category of drugs and help to understand their property. In addition, experienceddoctors had the probability of attending in-service trainings and different scientific conferences. Study also revealed that years of experience doctors and others health workers were associated with knowledge and attitude towards PV and ADR reporting[42, 43].

Doctors who claimed ADR reporting form exist in their hospital werealmostfour times more likely to report ADR cases as compared to those doctors who claimed that it didn’t exist in their hospital. The qualitative result also indicated that shortage of thereportingform in hospitals was contributed to under reporting of the ADR cases. This was in line with studies conducted inEthiopia and South India [19, 44].Study conducted in Uganda was also indicated the availability of ADR reporting is essential for ADR reporting[43] and the unavailability of the ADR reporting format is the common discouraging factor for ADR reporting[45].

Reporting ADR cases to MAH was significantly associated with ADR reporting practice. Doctors who responded as they were reported ADR cases encountered to the respective MAH were 21 times more likely to report ADR to the PV centreas compared to those doctors who opined as they did not report ADR to respective MAH. The reason might be due to MAH might have proper system to receive ADR reports[46] and informed the medical doctors on the existence of the national ADR reporting system including how, who, to whom and where to report ADR encountered.Another possible reason might be that MAH might provide feedbacks on the report and encouraged doctors to report. The finding was almost similar with a study conducted in Bulgaria[47].

## Strength and limitation of the study

The strength of the study was that quantitative data were triangulated with qualitative findings. Furthermore, the study considered factors such as specialization of doctors, structure and system of PV, linkage and support of hospital to/by PV center, and reporting of ADR cases to MAH. The study had also some limitations. Response bias among the doctors and key informants might affect the study findings. In addition, thequestioner used to assess the knowledge and attitude of doctors was not validated, despite it was tested before the actual data collection.

## Conclusion

Most doctors in Addis Ababa working in government hospitalsdid not report adverse drug reactions to the national medicine regulatory authority. Sex, level of education, work experience, existence of ADR reporting forms and ADR reporting to MAH were significantly associated with ADR reporting practice.

In addition, the common reasons for under reporting of adverse drug reactions among doctors were poor awareness of risk of under-reporting, lack of pre-service and in-service training, feeling that ADRis minor, absence of appropriate ADR reporting systems and tools, delay and/or absence of feedback on reported ADRs, over burden of doctors, negligence, fear of legal liability and communication gaps.

Therefore, it is important to aware doctors on the impact of a single report to ensure patients safety and train them on reporting system of the country. Moreover, concerted efforts need to be exerted to strengthen national pharmacovigilance system. It is also important to devise systemsto avail reporting tools including guidelines and reporting forms. The authority should also create efficientreportingsystem including electronic reporting system that encourage doctors to report any detected ADR cases so as to protect patients and clients.

## Abbreviations

ADR: Adverse Drug Reaction
DIC: Drug Information Center
DTC: Drug and Theraputic Committee
EFMHACA: Ethiopian Food, Medicine and Healthcare Administration and Control Authority
EPHI: Ethiopian Public Health Institute
HIV: Human Immunodeficiency Virus
MAH: Marketing Authorization Holder
PQM: Promoting the Quality of Medicines
PV: Pharmacovigilance
SPSS: Statistical Product and Service Solutions
USP: US Pharmacopeial Convention

## References

[1] FMHACA. Guideline for Adverse Drug Events Monitoring (Pharmacovigilance). 3rd Edition. Addis Ababa, Ethiopia; 2014.

[2] WHO. WHO guidelines on safety monitoring of herbal medicines in pharmacovigilance systems. Geneva: World Health Organization; 2004.

[3] Kongkaew C, Noyce PR, Ashcroft DM. Hospital admissions associated with adverse drug reactions: a systematic review of prospective observational studies. Annals of Pharmacotherapy. 2008;42(7–8):1017–25. 18594048 10.1345/aph.1L037

[4] Walter SR, Day RO, Gallego B, Westbrook JI. The impact of serious adverse drug reactions: a population‐based study of a decade of hospital admissions in New South Wales, Australia. British Journal of Clinical Pharmacology. 2017;83(2):416–26. 10.1111/bcp.13124 27614089 PMC5237693

[5] Angamo MT, Chalmers L, Curtain CM, Bereznicki LR. Adverse-drug-reaction-related hospitalisations in developed and developing countries: A review of prevalence and contributing factors. Drug Safety. 2016;39(9):847–57. 10.1007/s40264-016-0444-7 27449638

[6] Rolfes L, van Hunsel F, Taxis K, van Puijenbroek E. The impact of experiencing adverse drug reactions on the patient’s quality of life: a retrospective cross-sectional study in the Netherlands. Drug Safety. 2016;39(8):769–76. 10.1007/s40264-016-0422-0 27145946 PMC4933735

[7] WHO. WHO Pharmacovigilance Indicators: A practical manual for the assessment of pharmacovigilance systems. Geneva, Switzerland: World Health Organization; 2015 p. 1–10.

[8] Nisa Z, Zafar A, Sher F. Assessment of knowledge, attitude and practice of adverse drug reaction reporting among healthcare professionals in secondary and tertiary hospitals in the capital of Pakistan. Saudi Pharmaceutical Journal. 2018;26(4):453–61. 10.1016/j.jsps.2018.02.014 29844715 PMC5961757

[9] WHO. Safety monitoring of medical products: reporting system for the general public. Geneva, Switzerland: World Health Organization; 2012 p. 2–8.

[10] Khan SA, Goyal C, Chandel N, Rafi M. Knowledge, attitudes, and practice of doctors to adverse drug reaction reporting in a teaching hospital in India: An observational study. Journal of Natural Science, Biology, and Medicine. 2013;4(1):191–6. 10.4103/0976-9668.107289 23633861 PMC3633276

[11] Abideen SP. Practical implications of spontaneous adverse drug reaction reporting system in hospitals. Asian Journal Pharmceuticals and Clinical Research. 2013;6(4):10–5.

[12] John LJ, Arifulla M, Cheriathu J, Sreedharan J. Reporting of adverse drug reactions: a study among clinicians. Journal of Applied Pharmaceutical Science 2012;2(6):135–59.

[13] Iffat W, Shakeel S, Rahim N, Anjum F, Nesar S, Ghayas S. Pakistani physicians’ knowledge and attitude towards reporting adverse drug reactions. African Journal of Pharmacy and Pharmacology. 2014;8(14):379–85.

[14] Lemay J, Alsaleh FM, Al-Buresli L, Al-Mutairi M, Abahussain EA, Bayoud T. Reporting of adverse drug reactions in primary care settings in Kuwait: A Comparative study of physicians and pharmacists. International Journal of the Kuwait University, Health Science Centre. 2018;27(1):30–8. 10.1159/000487236 PMC5968250 29402876

[15] Berhe DF, Juhlin K, Star K, Beyene KG, Dheda M, Haaijer-Ruskamp FM, et al Adverse drug reaction reports for cardiometabolic drugs from sub-Saharan Africa: a study in VigiBase. Tropical Medicine andIinternational Hhealth. 2015;20(6):797–806. 10.1111/tmi.12481 25704305

[16] Ampadu HH, Hoekman J, de Bruin ML, Pal SN, Olsson S, Sartori D, et al Adverse drug reaction reporting in Africa and a comparison of individual case safety report characteristics between Africa and the rest of the world: Analyses of spontaneous reports in VigiBase®. Drug Safety. 2016;39(4):335–45. 10.1007/s40264-015-0387-4 26754924 PMC4796322

[17] Sabbleh GT, Akweongo P, Darko D, Dodoo ANO, Sulley AM. Adverse drug reaction reporting by doctors in developing country: A case study from Ghana. Ghana Medical Journal. 2016;48(4):189–93. 10.4314/gmj.v48i4.4 PMC4335427 25709133

[18] Abay SM, Dires T. Spontaneous adverse drug reaction reporting and the obstacle in Amhara Region Referral Hospitals, Ethiopia. Pharmacologyonline. 2008.

[19] Necho WM, Worku W. Assessment of knowledge, attitude and practice of health professionals towards adverse drug reaction reporting and factors associated with reporting. Journal of Pharmacovigilance. 2014;2:135.

[20] Belachew SA, Erku DA, Mekuria AB, Gebresillassie BM. Pattern of chemotherapy-related adverse effects among adult cancer patients treated at Gondar University Referral Hospital, Ethiopia: a crosssectional study. Drug, Healthcare and Patient Safety 2016;8:83–90. 10.2147/DHPS.S116924 27994485 PMC5153262

[21] Tatiparthi R, Mamo Y. Prevalence of ADRs and associated factors of antiretroviral treatment on HIV positive adults at Jush. Indian Journal of Pharmacy Practice 2014;7(4):8–15.

[22] EFMHACA. Guidelines for medicine registration In: 3rd Edition. Addis Ababa, Ethiopia. 2014.

[23] WHO. The importance of pharmacovigilance: Safety monitoring of medicinal products. 2002:1–48.

[24] Ermias A, Gurmesa G, Mesfin M, Mengistu A. Adverse drug reaction monitoring in Ethiopia: Analysis of case reports, 2002–2007. Ethiopian Journal of Health Development. 2011;2(168):168–73.

[25] Maigetter K, Pollock AM, Kadam A, Ward K, Weiss MG. Pharmacovigilance in India, Uganda and South Africa with reference to WHO's minimum requirements. International Journal of Health Policy and Management. 2015;4(5):295–305. 10.15171/ijhpm.2015.55 25905480 PMC4417633

[26] Rosli R, Ming LC. A Retrospective analysis of spontaneous adverse drug reactions reports relating to paediatric patients. 2016;11(6):e0155385. 10.1371/journal.pone.0155385 PMC4889073 27249414

[27] UMC. Safetymonitoring of medicinalproducts: Guidelines for setting up and running a pharmacovigilance centre,. the Uppsala Monitoring Centre (the UMC), WHO Collaborating Centre for International Drug Monitoring; 2000.

[28] CSA. 2007 Population and housing census of Ethiopia: Administrative Report. Addis Ababa: Central Statistical Authority; 2012.

[29] Tiruneh MA, Ayele BT. Practice of code of ethics and associated factors among medical doctors in Addis Ababa, Ethiopia. PloS one. 2018;13(8):e0201020 10.1371/journal.pone.0201020 30089133 PMC6082517

[30] Beyene KG, Beza SW. Self-medication practice and associated factors among pregnant women in Addis Ababa, Ethiopia. Tropical Medicine and Health. 2018;46:10 10.1186/s41182-018-0091-z 29743807 PMC5928590

[31] Bursac Z, Gauss CH, Williams DK, Hosmer DW. Purposeful selection of variables in logistic regression. Source Code for Biology and Medicine. 2008;3(1):17. 19087314 10.1186/1751-0473-3-17 PMC2633005

[32] Fadare JO, Enwere OO, Afolabi AO, Chedi BAZ, Musa A. Knowledge, attitude and practice of adverse drug reaction reporting among healthcare workers in a Tertiary Centre in Northern Nigeria. Tropical Journal of Pharmaceutical Research. 2011;10(3):235–42.

[33] Tew MM, Teoh BC, Mohd Baidi AS, Saw HL. Assessment of knowledge, attitude and practices of adverse drug reaction reporting among doctors and pharmacists in primary healthcare. Advances in Pharmacoepidemiology and Drug Safety. 2016;5(4):1–6.

[34] Thomas TM, Udaykumar P, Scandashree K. Knowledge, attitude and practice of adverse drug reaction reporting among doctors in a tertiary health care centre in South India. International Journal of Pharmacology and Clinical Sciences. 2013;2(3):82–8.

[35] Ajoy B, Kumar BB, Swapnanil G, Binita S. Knowledge, attitude and practices of pharmacovigilance among junior doctors of a tertiary health care institute in North East India. Scholars Journal of Applied Medical Sciences. 2016;4(9A):3248–53.

[36] Stergiopoulos S, Brown CA, Felix T, Grampp G, Getz KA. A Survey of adverse event reporting practices among US healthcare professionals. Drug Safety. 2016;39:1117–27. 10.1007/s40264-016-0455-4 27638657 PMC5045838

[37] Upadhyaya P, Seth V, Moghe VV, Sharma M, Ahmed M. Knowledge of adverse drug reaction reporting in first year postgraduate doctors in a medical college. Therapeutics and Clinical Risk Management. 2012;8:307–12. 10.2147/TCRM.S31482 22767994 PMC3387833

[38] Varallo FR, Guimarães SdOP, Abjaude SAR, Mastroianni PdC. Causes for the underreporting of adverse drug events by health professionals: a systematic review. Revista da Escola de Enfermagem da USP. 2014;48(4):739–47. 10.1590/s0080-623420140000400023 25338257

[39] Kamtane RA, Jayawardhani V. Knowledge, Attitude and perception of physicians towards adverse drug reaction (ADR) reporting: A Pharmacoepidemiological Study. Asian Journal Pharmacology and Clinical Research 2012;5(3):210–4.

[40] Leone S. 5-year trend of reporting adverse drug reaction: An Italian general practice experience. EC Pharmacology and Toxicology. 2017;5:29–37.

[41] Othman GQ, Ibrahim MIM, Alshakka M, Ansari M, Al-Qadasi F, Halboup AM. Knowledge and perception about pharmacovigilance among pharmacy students of Universities in Sana'a Yemen. Journal of Clinical and Diagnostic Research. 2017;11(6):Fc09–fc13. 10.7860/JCDR/2017/24228.10028 28764191 PMC5535384

[42] Kamal NN, Kamel EG, Mahfouz EM, editors. Adverse drug reactions reporting, knowledge, attitude and practice of physicians towards it in El Minia University Hospitals. International Public Health Forum; 2014;1(4):13–17.

[43] Katusiime B, Semakula D, Lubinga SJ. Adverse drug reaction reporting among health care workers at Mulago National Referral and Teaching hospital in Uganda. African Health Sciences. 2015;15(4):1308–17. 10.4314/ahs.v15i4.34 26958036 PMC4765406

[44] Ganesan S, Vikneswaran G, Reddy KC, Subrahmanyam DK, Adithan C. A Survey on knowledge, attitude and practice of pharmacovigilance towards adverse drug reactions reporting among doctors and nurses in a tertiary care hospital in South India. Journal of Young Pharmacists. 2016;8(4):471–6. 10.4103/0976-9668.210014 PMC5523529 28781488

[45] Gurmesa LT, Dedefo MG. Factors affecting adverse drug reaction reporting of healthcare professionals and their knowledge, attitude, and practice towards ADR reporting in Nekemte Town, West Ethiopia.BioMed Research International. 2016;2016:5728462 10.1155/2016/5728462 28042569 PMC5155121

[46] Singh A, Bhatt P. Comparative evaluation of adverse drug reaction reporting forms for introduction of a spontaneous generic ADR form. Journal of Pharmacology and Pharmacotherapeutics. 2012;3(3):228 10.4103/0976-500X.99417 23129957 PMC3487270

[47] Stoynova V, Getov IN, Naseva EK, Lebanova HV, Grigorov EE. Physicians’ knowledge and attitude towards adverse event reporting system and result to intervention–randomized nested trial among Bulgarian physicians. Medicinski Glasnik (Zenica). 2013;10(2):365–72. 23892860

